# Automated Diagnosis of Otitis Media: Vocabulary and Grammar

**DOI:** 10.1155/2013/327515

**Published:** 2013-08-07

**Authors:** Anupama Kuruvilla, Nader Shaikh, Alejandro Hoberman, Jelena Kovačević

**Affiliations:** ^1^Department of BME and Center for Bioimage Informatics, Carnegie Mellon University, Pittsburgh, PA 15213, USA; ^2^Division of General Academic Pediatrics, Children's Hospital of Pittsburgh, University of Pittsburgh School of Medicine, Pittsburgh, PA 15213, USA; ^3^Department of ECE Carnegie Mellon University, Pittsburgh, PA 15213, USA

## Abstract

We propose a novel automated algorithm for classifying diagnostic categories of otitis media: acute otitis media, otitis media with effusion, and no effusion. Acute otitis media represents a bacterial superinfection of the middle ear fluid, while otitis media with effusion represents a sterile effusion that tends to subside spontaneously. Diagnosing children with acute otitis media is difficult, often leading to overprescription of antibiotics as they are beneficial only for children with acute otitis media. This underscores the need for an accurate and automated diagnostic algorithm. To that end, we design a feature set understood by both otoscopists and engineers based on the actual visual cues used by otoscopists; we term this the *otitis media vocabulary*. We also design a process to combine the vocabulary terms based on the decision process used by otoscopists; we term this the *otitis media grammar*. The algorithm achieves 89.9% classification accuracy, outperforming both clinicians who did not receive special training and state-of-the-art classifiers.

## 1. Introduction

 Otitis media is a general term for middle-ear inflammation and may be classified clinically as either *acute otitis media *(AOM) or *otitis media with effusion *(OME); AOM represents a bacterial superinfection of the middle ear fluid and OME represents a sterile effusion that tends to subside spontaneously. Although middle ear effusion is present in both cases, this clinical classification is important because antibiotics are generally beneficial only for AOM [1, 2]. However, proper diagnosis of AOM as well as distinction from both OME and no effusion (NOE) requires considerable training (see Figure 1, e.g., images).

AOM is a frequent condition affecting the majority of the pediatric population for which antibiotics are prescribed. It is the most common childhood infection, representing one of the most frequent reasons for visits to the pediatrician. The number of otitis media episodes has increased substantially in the past two decades, with approximately 25 million visits to office-based physicians in the US and a total of 20 million prescriptions for antimicrobials related to otitis media yearly [3]. This results in significant social burden and indirect costs due to time lost from school and work, with an estimated annual medical expenditure of approximately 2$ billion [4].

The current standard of care in diagnosing AOM includes visual examination of the tympanic membrane with a range of available otoscopes: from simple hand-held ones with a halogen light source and low-power magnifying lens to more sophisticated, videootoscopes and otoendoscopes, which connect to a light source (halogen, xenon, or LED) and a computer and can record images or video. Single hand-held otoscopes do not permit acquisition of images and/or video and require diagnosis on the spot, while videootoscopes and otoendoscopes do; however, the clinician views the feed on a side screen while holding the device in the ear canal of an often-squirming young child.

*Misdiagnosis*. The inherent difficulties in distinguishing among the three diagnostic categories of otitis media, together with the above issues, make the diagnosis by nonexpert otoscopists notoriously unreliable and lead to the following

1. * Overprescription of Antibiotics*. AOM is frequently overdiagnosed; this happens when NOE or OME is diagnosed as AOM, resulting in unnecessary antibiotic prescriptions that lead to adverse effects and increased bacterial resistance [5]. Overdiagnosis is more common than underdiagnosis because doctors typically try to avoid the possibility of leaving an ill patient without treatment, leading to antibiotic prescriptions in uncertain cases.
2. * Underprescription of Antibiotics*. Misdiagnosis of AOM as either NOE or OME leads to underdiagnosis. Most importantly, children's symptoms are left unaddressed. Occasionally, underdiagnosis can lead to an increase in serious complications such as perforation of the tympanic membrane and, very rarely, mastoiditis [6].
3. * Increased Financial Costs and Burden*. There are direct and indirect financial costs associated with misdiagnoses such as medication costs, copayments, emergency department and primary care provider visits, missed work, and special day care arrangements.

For all the reasons above, accurate diagnosis is imperative to ensure that antimicrobial therapy is limited to the appropriate patients; this, in turn, increases the likelihood of achieving optimal outcomes and minimizing antibiotic resistance.

*Goal*. Currently, clinical diagnosis of otitis media is time consuming and subjective and shows limited intra and inter-observer reproducibility, underscoring the critical need for an accurate classification algorithm.

We develop the first such algorithm as diagnostic aid to classify tympanic membrane images into one of the three stringent clinical diagnostic categories: AOM, OME, and NOE.

To our knowledge, the only related work in this area is [7] where the authors investigate the influence of color on the classification accuracies of individual classes and conclude that the color alone is not sufficient for accurate classification.

*Guiding Principles. *To achieve our goal, we adopt the guiding principles below, partly inspired by [8–12]. The authors performed extensive research on understanding how humans perceive and measure similarity of color patterns. To understand and describe the mechanism of human perception, a subjective experiment was conducted, leading to a set of basic categories—*vocabulary* used by humans in judging similarity of color patterns and their relative importance and relationships, as well as the hierarchy of rules—*grammar*. We aim here to find the corresponding vocabulary and grammar of otitis media.

*(i) Vocabulary*. We aim to design a feature set understood by both otoscopists and engineers based on the actual visual cues used by otoscopists; we term this the *otitis media vocabulary*.

 To explore the diagnostic processes used, Dr. Shaikh et al. conducted a study to examine findings that the expert otoscopists use during their clinical diagnosis [13]. During the study, endoscopic still images of tympanic membranes of 783 children were obtained and examined by expert otoscopists. The examining otoscopist recorded information regarding a history of otalgia and findings concerning the following tympanic membrane characteristics: color (amber, blue, gray, pink, red, white, yellow), translucency (translucent, semiopaque, opaque), position (neutral, retracted, bulging), mobility (decreased, not decreased), and areas of marked redness, as distinct from mild or moderate redness (present, absent). A random sample of 135 (in ratio 2 : 2 : 1 of AOM : OME : NOE) of these images was sent for review to another group of 7 independent expert otoscopists, resulting in a dataset of 945 image evaluations. To control for differences in color rendition between computers, color-calibrated laptops were mailed to each expert. They were asked to independently describe tympanic membrane findings and assign a diagnosis of AOM/OME/NOE.

Just by evaluating still images, with no information about mobility or ear pain, the diagnosis (AOM versus no AOM) endorsed by the majority of experts was in agreement with the live diagnosis 88.9% of the time, underscoring the limited role that symptoms and mobility of the tympanic membrane have in the diagnosis of AOM. Live diagnosis refers to the diagnosis based on physical examination and evaluation of the child at the time of the encounter and is not based on images. Among both groups of otoscopists, bulging of the tympanic membrane was the finding judged best to differentiate AOM from OME: 96% of ears during live diagnosis and 93% of ear image evaluations were assigned a diagnosis of AOM. By members of the two groups who assigned the diagnosis of OME, bulging of the tympanic membrane was reported in 0% and 3% of ears during live diagnosis and ear image evaluations, respectively. Opacification of the tympanic membrane was the finding that best differentiated OME from NOE.

To design the otitis media vocabulary, we follow the guidelines in Table 1 that summarizes these otoscopic findings.

*(ii) Grammar*. We aim to design a rule-based decision process to combine the vocabulary terms based on the decision process used by otoscopists; we term this the otitis media grammar.

To design the grammar, we use the findings from [14], where the authors empirically examined the findings used by a group of expert otoscopists for diagnosing otitis media. In this study, relative importance of signs and symptoms in diagnosis of AOM was described and then used to develop a rule-based decision tree method to diagnose otitis media. At each visit of the patient, the otoscopist recorded the following tympanic membrane characteristics: color (amber, blue, gray, pink, white, yellow), degree of opacification (translucent, semi opaque, opaque), position (neutral, retracted, bulging), decreased mobility (yes, no), presence of air-fluid level(s) (yes, no), and presence of areas of marked redness (yes, no). A decision tree was then developed based on the recorded tympanic membrane characteristics using recursive partitioning to classify the cases into one of the three diagnostic categories. This manual decision tree uses two decisions to discriminate among the diagnostic categories; first, bulging is used to distinguish AOM from OME and NOE, and if no bulging was present, opacification or air-fluid level is used to distinguish between OME and NOE (see Figure 2). For ease of reference, we name the diagnosis of AOM, NOE, and OME as Stages 1, 2, and 3, respectively.

To design the otitis media grammar, we follow the guidelines in Figure 2 that summarizes this decision process.

*Validation*. We will compare our algorithm designed following the above guiding principles to diagnoses provided by three general pediatricians as well as five automated classifiers, three of which we designed previously and two generic classifiers that are available in the literature. The ground truth is provided by a panel of three expert otoscopists.

## 2. Methods

 A general classification algorithm consists of two parts: numerical feature extraction meant to discriminate among classes, and classification based on these features. In the otitis media classifier, we add a preprocessing step prior to feature extraction to minimize the impact of image artifacts.

### 2.1. Preprocessing

 To compute features, image preprocessing is crucial because it is expected that some regions may not be relevant or can contain foreign objects that occlude the tympanic membrane. Moreover, we aim to eliminate or minimize the impact of image artifacts associated with otoscopic images, which fundamentally consist of specular highlights. These artifacts will affect feature computation and hence must be corrected. To that end, we start with an automated segmentation step to locate the tympanic membrane and apply a local illumination correction to mitigate the problem of specular highlights. If a captured image is deemed not fit for processing, the algorithm will reject the image and prompt the clinician to retake it.

#### 2.1.1. Automated Segmentation of Tympanic Membrane

 Segmentation is a crucial step to extract relevant regions on which reliable features for classification can be computed. We now briefly summarize an active-contour based segmentation algorithm [15] we adapted for our purposes. First, a so-called snake potential of the grayscale version of the input image is computed, followed by a set of forces that outline the gradients and edges of the image. The active-contour algorithm [16] is then initialized by a circumference in the center of the image. The algorithm iteratively grows this contour and stops at a predefined convergence criterion, which leaves an outline that covers the relevant region in the image. This outline is used to generate the final mask that is applied to the input image to obtain the final result shown in Figure 3. We evaluated the performance of the algorithm on automatically segmented images against hand segmented images by expert otoscopists and found that we can automatically segment prior to classification without hurting the performance of the classifier. By adding this segmentation stage, the classification system becomes completely automated by not requiring the clinician to specify where the tympanic membrane is positioned.

#### 2.1.2. Correction of Specular Highlights

 One of the problems encountered is the presence of specular highlight regions caused by residual cerumen (wax) in the ear canal and wax on surface of the hair in the ear canal, which might remain after the examination. Cerumen reflects the light from the otoscope, which results in white regions in the image as shown in Figure 4 (top). These regions of local specular highlights have to be corrected.

Several methods [17–19] are shown to be robust in correcting local illumination changes. Most of these methods adjust the pixel intensity value of the image using a nonlinear mapping function for illumination correction based on the estimated local illumination at each pixel location and combining the adjusted illumination image with the reflectance image to generate an output image. The extent of possible image correction and editing ranges from replacement or mixing with another source image region to altering some aspects of the original image locally such as illumination or color. Since these methods can be used to locally modify image characteristics, our aim is to detect the specular highlights in the image and use these techniques to locally correct them. We use a simple thresholding scheme on image intensities to identify the specular highlight regions as shown in Figure 4 (middle row), followed by the Poisson image editing technique [19] to correct the identified regions in Figure 4 (bottom row).

#### 2.1.3. Rejection of Unreliable Data

 Some of the segmented images may contain large regions of white pixels due to overexposure. The above-mentioned techniques rely on using the neighboring pixels to approximate intensities in the region to be corrected and thus are effective when the region to be corrected is small. We empirically found that if the area of continuous white pixels is more than 15% of total pixels in the segmented tympanic membrane image, correcting such regions gives unreliable results and hence such an image should be rejected. Our aim is to use the rejection stage in the real application and prompt the clinician to retake the image until deemed suitable for processing.

### 2.2. Otitis Media Vocabulary

 The expert otoscopist uses his specialized knowledge when discriminating between the different diagnostic categories. The goal of our proposed methodology is to create a feature set—otitis media vocabulary, which will mimic the visual cues used by trained otoscopists.

#### 2.2.1. Methodology

 To design the otitis media vocabulary we will follow the process outlined in [20], where a histopathology vocabulary was designed for automated identification and delineation of tissues in images of H&E-stained teratomas. Similar vocabulary features were used in [21] for automated detection of colitis.

*Formulation of Initial Set of Descriptions*. We obtain initial descriptions of those characteristics best describing a given diagnostic category from the summary of otoscopic findings in Table 1.

*Computational Translation of Key Terms*. From this set, the key terms, such as *bulging*, are translated into their computational synonyms, creating a computational vocabulary. In our case, we construct a feature called bulging, which measures the area of the bulged region in the tympanic membrane.

*Computational Translation of Descriptions*. Using the computational vocabulary, the entire otoscopist's descriptions, such as *bulging and white*, are translated.

*Verification of Translated Descriptions*. Based on these translated descriptions, and without access to the image, the otoscopist tries to identify the diagnostic category being described, emulating the overall system with translated descriptions as features and the otoscopist as the classifier.

*Refinement of Insufficient Terms*. If the otoscopist is unable to identify a diagnostic category based on translated descriptions, or if a particular translation is not understandable, then that translation is refined and presented again to the otoscopist for verification.

*Otitis Media Vocabulary*. If the otoscopist is able to identify a diagnostic category based on translated descriptions, then the discriminative power of the key terms and their corresponding computational interpretations are validated, and these terms can be included as otitis media vocabulary terms to create features.

This feedback loop is iterated until a sufficient set of terms have been collected to formulate the otitis media vocabulary:

 bulging *f*_*b*_
 translucency *f*_*t*_
 central concavity *f*_*c*_
 amber level *f*_*a*_
 light *f*_*ℓ*_
 bubble presence *f*_bp_
 malleus presence *f*_*m*_
 grayscale variance *f*_*v*_

#### 2.2.2. Features

 We designed the vocabulary features, bulging, central concavity, malleus presence, translucency, amber level, and bubble presence based on otoscopic findings listed in Table 1. Supplementing the features designed based on otoscopic findings, we designed an additional two, light and grayscale variance, based on our observations and to catch classifier errors.

1. The first three vocabulary features, *bulging, central concavity, and light*, describe the distinct characteristics associated with AOM and will be used to construct Stage 1 of the grammar to identify AOM, mimicking Stage 1 in Figure 2. The vocabulary feature bulging is the exact computational translation of *bulging tympanic membrane* in Figure 2.
2. The next two vocabulary features, *malleus presence and translucency*, are indicative of NOE and will be used to construct Stage 2 of the grammar to identify NOE, mimicking Stage 2 in Figure 2. To describe *opacification* in Figure 2, we construct a vocabulary feature translucency, which detects the opposite.
3. The final three vocabulary features, *amber level, bubble presence, and grayscale variance*, describe the characteristics of OME and will be used to construct Stage 3 of the grammar to identify OME, mimicking Stage 3 in Figure 2. The vocabulary feature bubble presence is the exact computational translation of *air-fluid level* in Figure 2.

We now explain each of the vocabulary features in detail.

*Bulging*. In [14], the authors showed that bulging of the tympanic membrane is crucial for diagnosing AOM. We will design a feature that calculates the percentage of bulged region in the tympanic membrane; we call it the *bulging* feature. The goal is to derive a 3D tympanic membrane shape from a 2D image, by expressing it in terms of depth at each pixel. For example, in AOM, we should be able to identify high-depth variation due to bulging of the tympanic membrane in contrast to low-depth variation in NOE due to tympanic membrane being neutral or retracted. The shape from shading technique [22] can be applied to recover a 3D shape from a single monocular image. The input is a grayscale version of the segmented original RGB image *X* ∈ ℝ^*M*×*N*^ as shown in Figure 5(a). The depth at each pixel can be calculated in an iterative manner using the image gradient and a linear approximation of the reflectance function of the image.  Figure 5(b) shows the result of depth map *X*_*d*_ identifying the bulged regions in the tympanic membrane. The depth map *X*_*d*_ is then thresholded at *T*_*d*_ (here *T*_*d*_ = 0.6) to obtain a binary mask *X*_*b*_ of bulging regions in the tympanic membrane.

We then define the bulging feature as the mean of *X*_*b*_,

(1) $$\begin{array}{c} \;f_{b}=E\left[X_{b}\right]. \end{array}$$

*Central Concavity*. The tympanic membrane is attached firmly to the malleus that is one of the three middle ear bones called auditory ossicles. In the presence of an infection, the tympanic membrane begins to bulge in the periphery. The central region, however, remains attached to the malleus forming a concavity. We design a feature to identify the concave region located centrally in the tympanic membrane; we call it the *central concavity* feature. The input is a grayscale version (Figure 6(a)) of the segmented original RGB image *X* ∈ ℝ^*M*×*N*^ as in Figure 3. We use a sliding window to extract a local circular neighborhood, *X*_*R*_(*m*, *n*), of radius *R* (*R* = 60 in our experiments). That circular neighborhood is then transformed into its polar coordinates to obtain *X*_*R*_(*r*, *θ*), with *r* ∈ {1, 2,…, *R*}, *θ* ∈ [0, 2*π*], and

(2) $$\begin{array}{c} r=\;\sqrt{{\left(m-m_{c}\right)}^{2}+{\left(n-n_{c}\right)}^{2}},\;\;\theta =\arctan\frac{\left(n-n_{c}\right)}{\left(m-m_{c}\right)}, \end{array}$$

where (*m*_*c*_, *n*_*c*_) are the center coordinates of the neighborhood *X*_*R*_. In Figure 6(b), the resulting image has *r* as the horizontal axis and *θ* as the vertical one. The concave region changes from dark to bright from the center towards the periphery of the concavity; in polar coordinates this change from dark to bright occurs as the radius grows; see Figure 6(b). Defining the bright region *B* = {(*r*, *θ*) | *r* > *R*′} and the dark region *D* = {(*r*, *θ*) | *r* ≤ *R*′}, and with *R*′ ∈ [(1/4)*R*, (3/4)*R*], we compute the ratio of the two means,

(3) $$\begin{array}{c} f_{c,R^{\prime }}=\frac{E\left[X_{R}\left(r,\theta \right){|}_{(r,\theta )\in B}\right]}{E\left[X_{R}\left(r,\theta \right){|}_{(r,\theta )\in D}\right]}.\; \end{array}$$

As the concave region is always centrally located, we experimentally determine a square neighborhood *I* (here 151 × 151) to compute the central concavity feature,

(4) $$\begin{array}{c} f_{c}=\underset{\left(m,n\right)\in I,R^{\prime }}{\max}f_{c,R^{\prime }}. \end{array}$$

*Light*. Examination of the tympanic membrane is performed by an illuminated otoendoscope. The distinct bulging in AOM results in nonuniform illumination of the tympanic membrane, in contrast to the uniform illumination in NOE. Our aim is to construct a feature that will measure this nonuniformity as the ratio of the brightly lit to the darkly lit regions; we call it the *light* feature.

We start by performing contrast enhancement on the grayscale image in Figure 7(a) to make the nonuniform lighting prominent. The resulting image in Figure 7(b) is thresholded at *T*_*ℓ*_ (found experimentally) to obtain a mask of the brightly lit binary image *X*_bl_ in Figure 7(c).

To find the direction (*θ*_max⁡_) perpendicular to the maximum illumination gradient, we look at lines passing through (*m*_*c*_, *n*_*c*_) (the pixel coordinates at which *f*_*c*_ is obtained) at the angle *θ* with the horizontal axis. Defining the bright region *B* = {(*m*, *n*) | *n* ≥ tan(*θ*)(*m* − *m*_*c*_) + *n*_*c*_} and the dark region *D* = {(*m*, *n*) | *n* < tan(*θ*)(*m* − *m*_*c*_) + *n*_*c*_}, we compute the ratio of the two means,

(5) $$\begin{array}{c} r\left(\theta \right)=\frac{E\left[X_{\text{bl}}\left(m,n\right){|}_{(m,n)\in B}\right]}{E\left[X_{\text{bl}}\left(m,n\right){|}_{(m,n)\in D}\right]}. \end{array}$$

Then, the direction perpendicular to the maximum illumination gradient is given by

(6) $$\begin{array}{c} \theta_{\max}=\underset{\theta }{\arg\;\max}\;r\left(\theta \right) \end{array}$$

and we define the light feature as

(7) $$\begin{array}{c} f_{\ell }=r\left(\theta_{\max}\right). \end{array}$$

*Malleus Presence*. In OME or in NOE, the tympanic membrane position is either neutral or retracted and makes the short process of the malleus visible. We design a feature to detect the partial or complete appearance of the malleus that would help in distinguishing AOM from OME and NOE; we call it the *malleus presence* feature. To identify the presence of the malleus, we perform an ellipse fitting (shown as a red outline in Figure 8(a)) to identify the major axis. The image is then rotated to align the major axis with the horizontal axis. Mean-shift clustering [23] is then performed as shown in Figure 8(b), followed by Canny edge detection [24]. Hough transform [25] is applied on the obtained edges around the major axis (50-pixel neighborhood empirically obtained) to detect a straight line (shown in red Figure 8(c)) extending to the periphery that will indicate the visibility of the malleus. If such a line is detected then the feature malleus presence *f*_*m*_ is assigned a value of 1 and 0 otherwise.

*Translucency*. Translucency of the tympanic membrane is the main characteristic of NOE in contrast with opacity in AOM and semi-opacity in OME; it results in the clear visibility of the tympanic membrane, which is primarily gray. We design a feature to measure the grayness of the tympanic membrane; we call it the *translucency* feature. We do that by using a simple color-assignment technique. As these images were acquired under different lighting and viewing conditions, according to [26], at least 3–6 images are needed to characterize a structure/region under all lighting and viewing conditions. We take the number of images to be *N*_tl_ = 20.

To determine gray-level clusters in translucent regions, we extract *N*_*t*_ pixels from translucent regions (*N*_*t*_ = 100) of *N*_tl_ RGB images by hand segmentation to obtain a total of *N*_tl_ *N*_*t*_ pixels from images (here 2000). We then perform clustering of these *N*_tl_ *N*_*t*_ pixels using *k*-means clustering to obtain *K* cluster centers *c*_*k*_ ∈ ℝ^3^, *k* = 1, 2,…, *K*, (*K* = 10) capturing variations of gray in the translucent regions.

To compute the translucency feature for a given image *X*, for each pixel (*m*, *n*), we compute *K* Euclidean distances of *X*(*m*, *n*) to the cluster center *c*_*k*_, *k* = 1, 2,…, *K*,

(8) $$\begin{array}{c} d_{k}\left(m,n\right)=\sqrt{\;\sum_{i=1}^{3}{\left(X_{i}\left(m,n\right)-c_{k,i}\right)}^{2}}, \end{array}$$

with *i* = 1,2, 3, denoting the color channel. If any of the computed *K* distances falls below a threshold *T*_*t*_ = 10 (found experimentally), the pixel is labeled as translucent and belongs to the region *R*_*t*_ = {(*m*, *n*) | min⁡_*k*_ *d*_*k*_(*m*, *n*) < *T*_*t*_}. The binary image *X*_*t*_ is then simply the characteristic function of the region *R*_*t*_, *X*_*t*_ = *χ*_*R*_*t*__.

We then define the translucency feature as the mean of *X*_*t*_,

(9) $$\begin{array}{c} f_{t}=E\left[X_{t}\right]. \end{array}$$

*Amber Level*. We use the knowledge that OME is predominantly amber or pale yellow to distinguish it from AOM and NOE. We design a feature to measure the presence of amber in the tympanic membrane; we call it the *amber* feature. We apply a color-assignment technique similar to that used for computing *X*_*t*_ to obtain a binary image *X*_*a*_, indicating amber and nonamber regions. We define the amber feature as the mean of *X*_*a*_,

(10) $$\begin{array}{c} f_{a}=E\left[X_{a}\right]. \end{array}$$

*Bubble Presence*. The presence of visible air-fluid levels, or bubbles, behind the tympanic membrane is an indication of OME. We design a feature to detect the presence of bubbles in the tympanic membrane; we call it the *bubble presence* feature. The algorithm takes in red and green channels of the original RGB image and performs Canny edge detection [24], to place parallel boundaries on either sides of the real edge, creating a binary image *X*_bp_ in between. This is followed by filtering and morphological operations to enhance edge detection and obtain smooth boundaries. We then define the bubble feature as the mean of *X*_bp_,

(11) $$\begin{array}{c} f_{\text{bp}}=E\left[X_{\text{bp}}\right]. \end{array}$$

*Grayscale Variance*. Another discriminating feature is the variance of the intensities across the grayscale version of the image *X*_*v*_. We define the feature *grayscale variance* as the variance of the pixel intensities in the image *X*_*v*_,

(12) $$\begin{array}{c} f_{v}=\text{var}\left(X_{v}\right); \end{array}$$

for example, OME has a more uniform appearance than AOM and NOE and has consequently a much lower variance that can be used to distinguish it from the rest.

### 2.3. Otitis Media Grammar

 The modeling of human perception of otitis media diagnosis is new—starting with the vocabulary feature design and the set of rules considered as the basic grammar of the otoscopist's language. For the actual implementation of the grammar, it is important to understand the way these rules are applied. An important aspect of our work is to use feedback from expert otoscopists to improve classification performance by trying to mimic their diagnostic process.

In our previous work [27], we designed an initial grammar as a simple hierarchical classifier that uses two levels. At the first level, binary decisions were used to split the images into two superclasses; AOM/OME (acute infection/middle ear fluid infection) and NOE/OME (no infection/middle ear fluid infection). At the second level, these superclasses were split into individual diagnostic categories using a weighted combination (*w*_*a*_, *w*_bp_, *w*_*t*_, *w*_*v*_) of four features, amber level *f*_*a*_, bubble presence *f*_bp_, translucency *f*_*t*_, and grayscale variance *f*_*v*_. The weighted combination *w*_*a*_ *f*_*a*_ + *w*_bp_ *f*_bp_ + *w*_*t*_ *f*_*t*_ + *w*_*v*_ *f*_*v*_ was used to split superclasses into AOM/OME/NOE.

Here, we design the grammar to mimic the decision process used by expert otoscopists in Figure 2 exactly. The decision process will use a hierarchical rule-based classification scheme based on the domain knowledge of the expert otoscopists. The classification is done in three stages by distinguishing one diagnostic category at a time: AOM (Stage 1), NOE (Stage 2), and OME (Stage 3), respectively, which we now describe in more detail.

#### 2.3.1. Stage 1: Identification of AOM

 As the first stage, we detect the instances of AOM based on bulging, light, central concavity, and malleus presence features as shown in Figure 9. While ideally, if there is bulging present, the image should be classified as AOM as in Figure 2, our bulging feature alone cannot accomplish the task; we use the other features in the otitis media vocabulary that describe the AOM characteristics such as light, central concavity, and malleus presence to aid separation of AOM from NOE and OME. In some cases, OME images can exhibit partial bulging and therefore have a high possibility of being grouped as AOM. In such cases, we use low amber level to distinguish AOM from OME.

#### 2.3.2. Stage 2: Identification of NOE

 Low values of bulging, light, central concavity, and malleus presence features eliminate the possibility of AOM being the diagnosis. Such a situation results in either the diagnosis being NOE or OME (see Figure 10). In Stage 2, our goal is to distinguish NOE from OME. The translucency feature, which is the most distinguishing characteristic of NOE, can be used here to identify normal cases. A high value of translucency clearly indicates NOE and low values of those features characteristic of OME indicate NOE. Thus, in this stage, NOE is identified from the superclass NOE/OME by a high value of the translucency feature or low values of all the features characteristic of OME: amber level, bubble presence, and grayscale variance.

#### 2.3.3. Stage 3: Identification of OME

Figure 11 shows the complete otitis media grammar. Most of the OME cases are identified from the superclass NOE/OME from Stage 2 as high values of amber level, bubble presence, and grayscale variance features. Some cases of OME can exhibit partial bulging resulting in high values of the bulging feature; in such cases, we can correctly detect OME if the values of light and central concavity features are low, and the value of amber level feature is high.

The threshold values for the features were calculated during the training phase of the algorithm. We performed a fivefold nested cross-validation. During each fold, the data was split into training and testing, and the training set was further split into two sets: learning and validation. We used misclassification rate on the validation set as the criterion to learn the threshold for each split. The threshold was fixed where we obtained the least misclassification rate during training and was used on the testing set.

The complete otitis media grammar we designed in Figure 11 thus follows the exact structure of the decision tree designed by expert otoscopists in Figure 2.

## 3. Results and Discussion

### 3.1. Ground Truth: Diagnosis by Expert Otoscopists

 As part of a clinical trial evaluating the efficacy of antimicrobials in young children with acute otitis media, 826 tympanic membrane images were collected using an otoendoscope from children with AOM, OME, and NOE [28]. A panel of three expert otoscopists examined these images and provided labels. As these images pose challenges even for expert otoscopists, the agreement was rather poor in labeling the images. Having accurate ground-truth labels is crucial for algorithm development, and thus, we asked the panel to rediagnose the entire dataset while also providing a diagnosis confidence level for each image; levels between 80and100 indicated high confidence in diagnosis, while levels below 30 indicated almost no confidence in diagnosis. Based on these, we selected a subset for which the three experts gave the same diagnosis and expressed confidence of over 60 in that diagnosis; we use this set as our *ground truth* set. The number of images in this ground-truth set is 181; 63 AOM, 70 OME, and 48 NOE. It is important to note that even for these highly trained expert otoscopists, this set is a challenging one, as there were no acquisition guidelines, and thus, the images depict a diverse set of conditions.

### 3.2. Validation: Diagnosis by General Pediatricians

 To validate the algorithm against a realistic diagnostic situation, we asked three general pediatricians to examine our ground-truth set of 181 tympanic membrane images provided by expert otoscopists. The experiment also required them to state their level of confidence in diagnosing each of the tympanic membrane images. In cases of diagnosis with high confidence, the examiner assigned only one diagnostic category to the image, whereas in cases where the confidence of diagnosis was either medium or low, the examiner was asked to also provide a second possible choice of diagnosis, resulting in two diagnoses of an image representing first and second diagnostic choices, respectively.

To evaluate how the group of three general pediatricians performed on the ground-truth dataset, Table 2 shows three confusion matrices: the first is the average diagnosis by the three pediatricians, while the other two are average diagnoses with high and medium/low confidence, respectively. The diagnostic accuracy that was obtained as an average of the accuracies from the three examining pediatricians was found to be 79.6% (91.7%, 75.7%, and 71.3%, resp.), well below that of expert otoscopists that we use as our ground truth of 100%.

In terms of misdiagnoses, NOE and OME are the categories with the highest level of misdiagnosis. The misdiagnosis of OME as AOM (15.7%) is clearly a cause of concern since it leads to the unnecessary prescription of antibiotics. Similarly, NOE is often misdiagnosed as OME (45.8%). It is surprising to note that only 50% cases of NOE were diagnosed with high confidence, of which 9 out of 24 were misdiagnosed. In the remaining 50% of cases, 13 out of 24 (54.2%) were misdiagnosed as OME; such instances of misdiagnosis may lead to unnecessary treatment procedures.

### 3.3. Validation: Automated Classifiers

 To validate our algorithm, we also compare it to five automated classifiers, three of which we designed previously, correlation filter classification system, multiresolution classifier and SIFT and shape description using SVM classifier, and two that are available in the literature, WND-CHARM classifier and random forest classifier. We now briefly describe each of these. Note that for all the experiments, we used a 5-fold cross-validation setup.

#### 3.3.1. Correlation Filter Classification System

 In this classifier, the image is first transformed into the polar domain. Overlapping concentric annular regions of different radii are extracted from the image. The center of the annular regions is assigned as the centroid of the segmented tympanic membrane image. During the training phase, templates of annular regions for each class are obtained. These templates are then used to assign a class label to the test images based on their similarity using normalized cross-correlation measure.

#### 3.3.2. Multiresolution Classifier

 The multiresolution classifier, which was designed for biomedical applications [29], decomposes the image into subbands using a multiresolution decomposition (e.g., wavelets or wavelet packets), followed by feature extraction and classification in each subband using neural networks (any classifier can be used in each individual subband) and a global decision based on weighted individual subband decisions. We ran the multiresolution classifier with 2 levels and 26 Haralick texture features on the grayscale image and each of the 20 subbands (546 in total).

#### 3.3.3. SIFT and Shape Descriptors with SVM Classifier

 In this classifier, we combined SIFT and shape features. SIFT features [30, 31] are first extracted from the images using the VLFeat library [32]. The shape descriptors were used as an attempt to detect bulging in the tympanic membrane. The main idea was to extract areas with bright and dark symmetry. On the segmented image, we applied phase symmetry detection algorithm described in [33]. Bright and dark regions were segmented using Otsu thresholding algorithm [34], resulting in two masks; one for the bright bulging regions and the other for the rest. Based on these masks the following features were computed: total area of bright regions, total area of dark regions, average symmetry measure in bright areas, number of dark regions, number of bright regions, and mean area of bright regions. All these features were normalized and we used a bag-of-words model. The classification was performed using support vector machine [35].

#### 3.3.4. WND-CHARM Classifier

 This is a universal classifier that extracts a large number (4,008) of generic image-level features [36]. The computed features include polynomial decompositions, high contrast features, pixel statistics, and textures. These features are derived from the raw image, transforms of the image, and compound transforms of the image (transforms of transforms). The algorithm performs a feature selection during the training stage by assigning a weight to each feature depending on its ability to distinguish between the classes. These weighted features are then used to classify test images based on their similarity to the training classes using the nearest neighbor algorithm.

#### 3.3.5. Random Forest Classifier

 This is an ensemble classifier [37] that consists of many decision trees and outputs the class that is the result of the majority vote of the classes output by individual trees. Each split was based on the feature from the randomly selected subset of 5 features that gave the best performance. The number of trees in the forest is fixed as 500 since during multiple runs of random forest we observed that the out-of-bag error converged in the range of 475–500 trees. We used the implementation of random forest in [38].

### 3.4. Results

Table 3 compares the performance of the diagnosis by three general pediatricians (GP) as well as eight classifiers: correlation filter classification system (CFC), WND-CHRM (WCM), multiresolution classifier (MRC), SIFT and shape descriptors with SVM classifier (SSC), random forest classifier (RF), our previous classifier from [27], and otitis media classifier (OMC).  Table 4 compares the results of the above-mentioned classifiers on the dataset of 170 images after automatic rejection of unreliable images. For ease of reference we name the otitis media classifier with rejection as OMCR. The otitis media classifier with rejection outperforms the other classifiers by a fair margin (12.8%). Random forest classifier shows the highest performance among the five compared algorithms but fails to outperform the otitis media classifiers. There are a couple of reasons for this poorer performance: since each image is assigned an output label based on majority vote of outputs from all the decision trees in the forest, the final output label can be a result contributed by poorly formed decision trees, and, a random forest classifier is known to exhibit better performance when the features used are uncorrelated which is not the case in this work, since more than one vocabulary feature is directly targeted to characterize a specific diagnostic category.

 While the overall performance increases between the otitis media classifier presented in [27] and otitis media classifier using the new vocabulary and grammar might not seem substantial, the increase in classification accuracy of AOM cases is significant. This increase can be attributed to the new grammar presented in Figure 11, which includes new vocabulary features; bulging and malleus presence. In [27], identifying AOM was solely based on central concavity and light features, which only indicate the presence of a bulge unlike the bulging feature that measures the total area of bulging in the tympanic membrane. The performance presented by otitis media classifier with rejection is a trade-off between misclassification and not classifying all the input data. A total of 11 (2 AOM, 3 OME, and 6 NOE) images were rejected using a simple rejection procedure explained earlier. We believe that this rejection step during preprocessing will ensure the collection of good-quality images that are suitable for processing and high-quality diagnosis.

Overall, the otitis media classifier performs better than the average of the three general pediatricians by a good margin (from 79.6% to 85.6%). Note that for the comparison to be fair, we did not compare the performance of the pediatricians to the otitis media classifier with rejection because they do not have an objective way of rejecting images of poor quality. At the same time, the rejection capability is a clear advantage of an automated algorithm and leads to improved performance (from 85.6% without rejection to 89.9% with rejection). Pediatricians performed well on diagnosing AOM but with a high possibility of overdiagnosing AOM. When comparing misdiagnoses of OME and NOE as AOM between pediatricians and the algorithm, 15.7% (11 out of 70) cases of OME and 8.3% (4 out of 48) cases of NOE were misdiagnosed as AOM by pediatricians compared to 10.0% (7 out of 70) cases of OME and 4.2% (2 out of 48) of NOE by the classifier, with a *P*  value of 0.1309 for the two-tailed Fisher exact test. When comparing misdiagnoses of NOE and OME between pediatricians and the algorithm, 50.0% (24 out of 48) cases of NOE were misdiagnoses as OME by pediatricians compared to only 14.6% (7 out of 48) by the classifier, with a *P* value of 0.0016 for the two-tailed Fisher exact test. From these observations, we conclude that, on average, our algorithm outperforms general pediatricians.

The above discussion validates our methodology that a small number of targeted, physiologically meaningful features, vocabulary, together with a well-designed grammar that mimics the decision process of expert otoscopists, is what is needed to achieve accurate classification in this problem.

## 4. Conclusions and Future Work

 We created an automated system for classifying the three diagnostic categories of otitis media. Our guiding principles were to design a *vocabulary* of features that mimics the actual visual cues used by expert otoscopists as well as a *grammar* to mimic their decision-making process. This automated algorithm that exhibits high levels of accuracy in identifying the diagnostic categories of otitis media is comparable to the diagnoses by expert otoscopists.

Results demonstrate that our simple and concise 8-feature otitis media vocabulary is effective on the problem, underscoring the importance of using targeted, physiologically meaningful features instead of a large number of general-purpose features. The classification process, grammar, is a hierarchical process mimicking the diagnostic process used by otoscopists. Increasing the accuracy from the current stage becomes harder, as we have reached a high accuracy range; we now discuss potential strategies for achieving that as directions for further work.

Images captured using a digital otoscope exhibit a large variability. Depending on the angle and amount of light incident on the membrane and the ear canal, we encounter different illumination problems related to brightness and contrast. In our current implementation, we only correct local illumination problems but have not solved for global illumination problems. Artifacts such as shading, shadows, and changes due to global variation in the intensity or color due to overexposure or underexposure will affect feature computation. Strategies for minimizing such artifacts are subject of future studies. In [39], the illumination field is estimated from an image and then compensated by using the estimated field and thus recovering the reflectance function. Global illumination correction can also be achieved as shown in [40] where the algorithm uses both illumination field and surface reflectance. The algorithm reports good performance in case of sufficient illumination variation as well as surface reflectance on natural scene images. These methods have also been applied in other image classification problems before feature extraction. We have not explored the issue of illumination normalization and plan to do so in future work.

Another problem we have observed has to do with the natural variability across the images, perceived as different orientations or poses of the tympanic membrane resulting from nonuniform positioning of the otoscope while capturing the image. If deemed necessary to deal with the variability of pose, we will investigate the use of active appearance models [41–43], which have been successfully used to register face images from an arbitrary pose to a frontal face or other specified profiles [44]. This preprocessing is implemented before feature extraction; it aligns new images to a universal template, with the aim of making feature extraction more stable. In other image recognition fields, such as in face recognition, the use of image preprocessing to align and normalize images is crucial for the overall performance of the system and might provide a performance boost in our case as well.

Finally, we believe that the performance of our system could be further improved by refining the otitis media grammar. Although the current grammar has a set of clear intuitive rules closely mimicking the expert otoscopists, we intend to use soft decision splits on each feature instead of hard binary decisions. Our current method does not use the relative importance of vocabulary feature in decision making. Future work includes investigating methods for refining the hierarchical decision process and using the relative importance of each of the vocabulary features in the classification process.

We expect to establish that the diagnostic categories of otitis media or its absence that are provided by our automated otitis media classification system are accurate representations of what expert otoscopists diagnose when examining the images of tympanic membranes. Should the otitis media classifier demonstrate good diagnostic capability, it can be employed as a clinical diagnostic aid to drastically decrease both underdiagnosis and overdiagnosis of AOM, assuring adequate antimicrobial use when AOM is present and reducing inappropriate use when AOM is absent, thus avoiding adverse side effects and the risk of contributing to bacterial resistance.

## Figures and Tables

**Figure 1:**
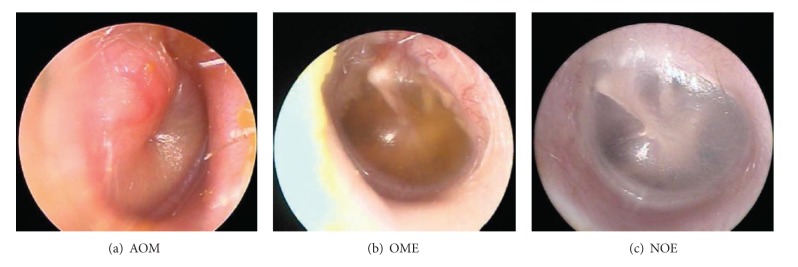
Sample (cropped) images from the three diagnostic categories of otitis media.

**Figure 2:**
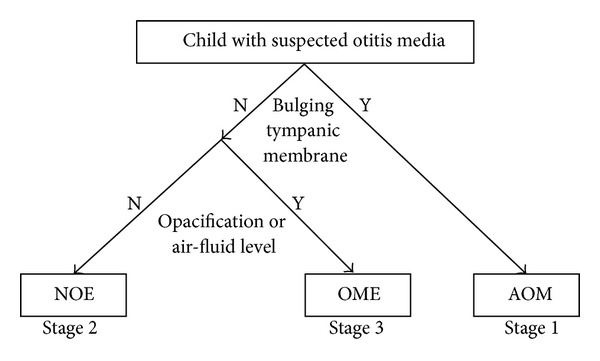
Guidelines for grammar design: decision tree for the diagnosis of otitis media [14].

**Figure 3:**
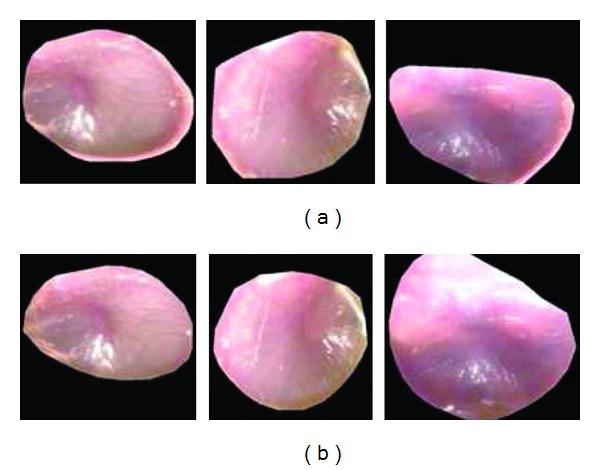
Comparison of automated segmentation (a) and hand segmentation by expert otoscopists (b).

**Figure 4:**
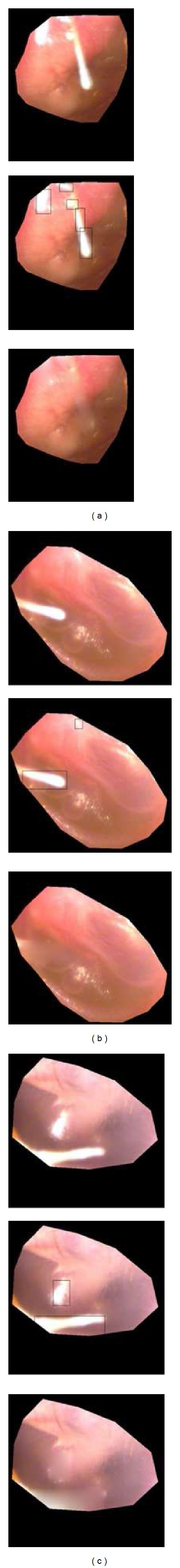
Correction of specular highlights for AOM (a), OME (b), and NOE (c). Input images are in the top row, identification of specular highlight regions in the middle row, and correction of the identified regions in the bottom row.

**Figure 5:**
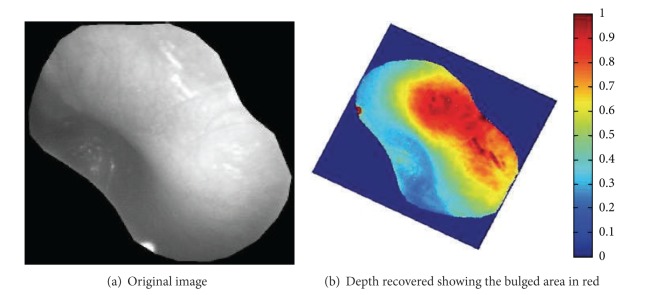
Computation of the bulging feature.

**Figure 6:**
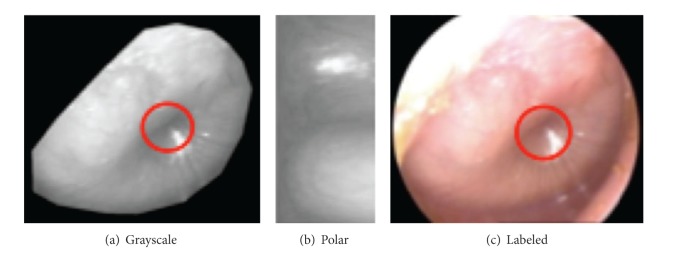
Computation of the central concavity feature.

**Figure 7:**
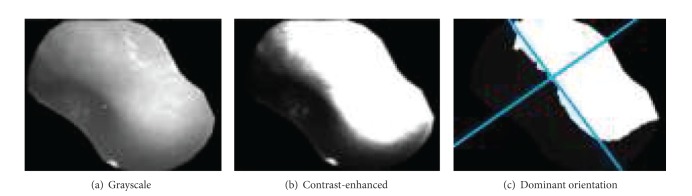
Computation of the light feature.

**Figure 8:**
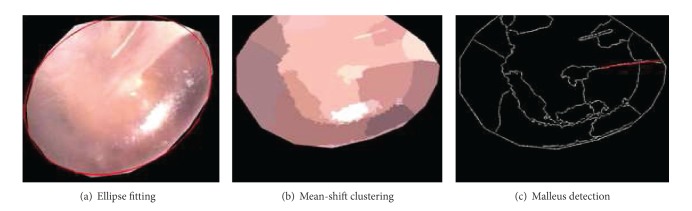
Computation of the malleus presence feature.

**Figure 9:**
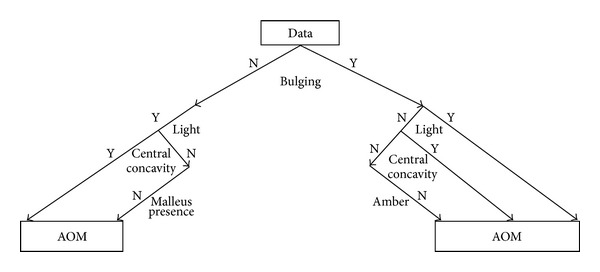
Stage 1: grammar for identifying AOM.

**Figure 10:**
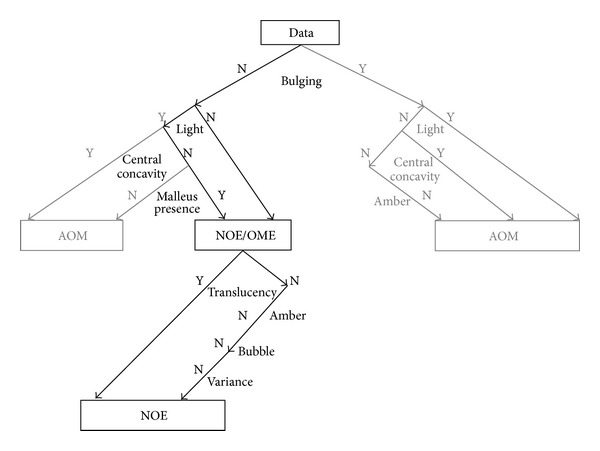
Stage 2: grammar for identifying NOE (black arrows/boxes denote those paths belonging to this stage; gray ones belong to Stage 1).

**Figure 11:**
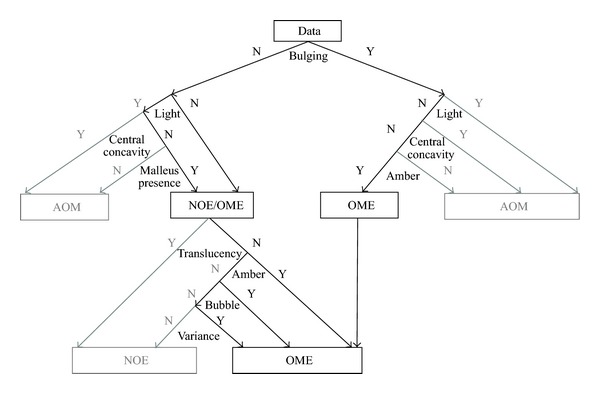
Stage 3: grammar for identifying OME (black arrows/boxes denote those paths belonging to this stage; gray ones belong to Stages 1 and 2).

**Table 1:** Guidelines for vocabulary design: Otoscopic findings associated with clinical diagnostic categories of tympanic membrane images [13].

	AOM	OME	NOE
Color	White, pale yellow, markedly red	White, amber, gray, blue	Gray, pink
Position	Distinctly full, bulging	Neutral, retracted	Neutral, retracted
Translucency	Opacified	Opacified, semiopacified	Translucent

**Table 2:** Diagnoses by three general pediatricians (columns) versus the ground truth of expert otoscopists (rows).

	Total			High confidence			Medium/low confidence		
	AOM	OME	NOE	AOM	OME	NOE	AOM	OME	NOE
AOM	62	1	0	60	0	0	2	1	0
OME	11	56	3	6	37	1	5	19	2
NOE	4	18	26	1	8	15	3	10	11

Accuracy	**79.6**%								

**Table 3:** Classification accuracies (in %) on the ground-truth set of 181 tympanic membrane images. Each row corresponds to the class-wise classification accuracies and columns correspond to the diagnosis by three general pediatricians (GP) as well as the following algorithms: correlation filter classification system (CFC), WND-CHRM (WCM), multiresolution classifier (MRC), SIFT and shape descriptors with SVM classifier (SSC), random forest classifier (RF), our previous classifier from [27], and otitis media classifier (OMC).

	CFC	WCM	MRC	SSC	GP	RF	[27]	OMC
AOM	66.7	68.2	53.5	66.7	98.4	84.1	81.3	88.8
OME	57.1	60.8	66.3	81.0	80.0	81.4	85.7	82.6
NOE	62.5	63.4	75.1	60.0	54.2	66.6	81.4	85.4

Accuracy	61.8	64.1	64.1	70.2	79.6	80.1	84.0	**85.6**

**Table 4:** Classification accuracies (in %) on the ground-truth set of 170 tympanic membrane images out of 181 images after rejection. Each row corresponds to the class-wise classification accuracies and columns correspond to classification by the following algorithms: correlation filter classification system (CFC), WND-CHRM (WCM), multiresolution classifier (MRC), SIFT and shape descriptors with SVM classifier (SSC), random forest classifier (RF), and otitis media classifier with rejection (OMCR).

	CFC	WCM	MRC	SSC	RF	OMCR
AOM	65.6	65.5	61.0	76.2	80.3	90.0
OME	56.7	58.2	65.3	72.8	79.1	89.1
NOE	71.4	69.0	83.9	58.3	69.4	93.2

Accuracy	63.6	63.6	68.2	70.0	77.1	**89.9**
